# The application value of deep learning-based nomograms in benign–malignant discrimination of TI-RADS category 4 thyroid nodules

**DOI:** 10.1038/s41598-024-58668-6

**Published:** 2024-04-03

**Authors:** Xinru Zhang, Cheng Jia, Meng Sun, Zhe Ma

**Affiliations:** 1 Department of Medical Ultrasound, The First Affiliated Hospital of Shandong First Medical University & Shandong Provincial Qianfoshan Hospital, Shandong Medicine and Health Key Laboratory of Abdominal Medical Imaging, Jinan, 250014 China; 2 Department of Radiology, The Second Affiliated Hospital of Shandong First Medical University, Taian, 271000 China

**Keywords:** Diseases, Health care, Medical research, Oncology, Engineering

## Abstract

Thyroid nodules are a common occurrence, and although most are non-cancerous, some can be malignant. The American College of Radiology has developed the Thyroid Imaging Reporting and Data System (TI-RADS) to standardize the interpretation and reporting of thyroid ultrasound results. Within TI-RADS, a category 4 designation signifies a thyroid nodule with an intermediate level of suspicion for malignancy. Accurate classification of these nodules is crucial for proper management, as it can potentially reduce unnecessary surgeries and improve patient outcomes. This study utilized deep learning techniques to effectively classify TI-RADS category 4 thyroid nodules as either benign or malignant. A total of 500 patients were included in the study and randomly divided into a training group (350 patients) and a test group (150 patients). The YOLOv3 model was constructed and evaluated using various metrics, achieving an 84% accuracy in the classification of TI-RADS category 4 thyroid nodules. Based on the predictions of the model, along with clinical and ultrasound data, a nomogram was developed. The performance of the nomogram was superior in both the training and testing groups. Furthermore, the calibration curve demonstrated good agreement between predicted probabilities and actual outcomes. Decision curve analysis further confirmed that the nomogram provided greater net benefits. Ultimately, the YOLOv3 model and nomogram successfully improved the accuracy of distinguishing between benign and malignant TI-RADS category 4 thyroid nodules, which is crucial for proper management and improved patient outcomes.

## Introduction

In recent years, the detection rate of thyroid nodules has increased, leading to a rise in the incidence of thyroid cancer^1^. Ultrasound imaging has become the preferred diagnostic method for evaluating thyroid nodules due to its non-invasive, radiation-free, real-time, and repeatability^2,3^. However, accurately differentiating between benign and malignant thyroid nodules through ultrasound remains challenging. Moreover, the interpretation of ultrasound results may vary among practitioners, influenced by their experience and professional skill^4^.

In clinical practice, TI-RADS category 4 thyroid nodules pose a challenge for sonographers. These nodules can be further classified into subtypes 4a, 4b, and 4c, each requiring different interventions. Accurately classifying these nodules is critical to avoid unnecessary biopsies and surgeries^5^. The subjective nature of ultrasound interpretation and varying levels of diagnostic experience can result in the misclassification of nodules that should be categorized as TI-RADS 3 or 5 as category 4. This misclassification can lead to unnecessary biopsies and surgeries, causing patient discomfort and increasing healthcare costs.

In recent times, the field of artificial intelligence (AI) has witnessed significant advancements in the diagnosis of thyroid nodules through ultrasound imaging. AI techniques, such as deep learning algorithms, have shown great promise in enhancing automated diagnosis capabilities and aiding healthcare professionals in accurately distinguishing between benign and malignant thyroid nodules^6^. Given the challenges of accurately diagnosing TI-RADS category 4 thyroid nodules and the potential risks associated with misclassification, there is a need to explore new methods to improve diagnostic accuracy. Meanwhile, recent advances in AI have shown great potential in improving the accuracy and efficiency of medical diagnosis, especially in the field of ultrasound medicine^7,8^.

In this study, deep learning-based column line drawings were developed to effectively differentiate between benign and malignant cases in patients with TI-RADS category 4. This provides clinicians with a more informed decision-making process, reducing unnecessary biopsies and surgeries and providing a valuable tool for patient care.

## Results

### Comparison of baseline characteristics

A total of 500 patients with 500 TI-RADS category 4 thyroid nodules were included in this study. The patients’ ages ranged from 23 to 78 years, with a mean age of 55 (40–66) years. Among the patients, 219 (43.8%) were female and 281 (56.2%) were male. Based on pathological findings, 223 (44.4%) nodules were classified as benign and 277 (55.6%) as malignant. The study population was divided into a training group, consisting of 205 men and 145 women with a mean age of 55 (40–66) years, and a test group, consisting of 76 men and 74 women with a mean age of 54 (41–65) years.

Statistical analysis of the basic clinical and ultrasound data revealed significant differences (*P* < 0.05) between patients with malignant and benign nodules in both the training and test groups. These differences included age, maximum diameter of thyroid nodules, gender, and TI-RADS grading. Detailed information can be found in Table 1.

**Table 1:** Comparison of basic information on benign and malignant nodules in the training and test groups.

Projects	Training group (n = 350)				Test group (n = 150)			
	Malignant (n = 198)	Benign (n = 152)	Wilcoxon two-sample rank sum test		Malignant (n = 79)	Benign (n = 71)	Wilcoxon two-sample rank sum test	
			*Z*	*P*			*Z*	*P*
Age	63.0 (49.0, 72.0)	46.0 (38.0, 57.0)	7.28	< 0.001	56.0 (47.5, 69.5)	46.0 (38.0, 59.0)	3.40	0.001
Diameter of the nodule	8.3 (6.6, 12.7)	10.0 (7.0, 13.0)	2.14	0.033	8.0 (6.5, 12.5)	9.8 (7.4, 12.0)	0.93	0.035

	Malignant (n = 198)	Benign (n = 152)	Chi-square test		Malignant (n = 79)	Benign (n = 71)	Chi-square test	
			*χ*^2^	*P*			*χ*^2^	*P*
Gender								
Male	133 (64.9%)	72 (35.1%)	13.9	< 0.001	47 (61.8%)	29 (38.2%)	5.2	0.023
Female	65 (44.8%)	80 (55.2%)			32 (43.2%)	42 (56.8%)		
TI-RADS classification								
TI-RADS 4a	36 (28.1%)	92 (71.9%)	79.5	< 0.001	13 (24.1%)	41 (75.9%)	31.9	< 0.001
TI-RADS 4b	75 (62.0%)	46 (38.0%)			30 (58.8%)	21 (41.2%)		
TI-RADS 4c	87 (86.1%)	14 (13.9%)			36 (80.0%)	9 (20.0%)		

Univariate analysis revealed that age, maximum diameter of thyroid nodules, gender, and TI-RADS grading significantly influenced the results in both the training and testing groups (*P* < 0.05). However, in logistic regression analyses, age, maximum diameter of thyroid nodules, gender, and TI-RADS grading were found to have a significant impact on the results in the training group (*P* < 0.05). In the test group, only age and TI-RADS grading remained significant predictors in the model (*P* < 0.01), indicating their importance in outcome prediction. These findings are summarized in Table 2. In conclusion, this study emphasizes the significance of age and TI-RADS grading in predicting outcomes and provides valuable insights for future research. Therefore, incorporating age and TI-RADS grading factors into the model can enhance the accuracy of outcome prediction.

**Table 2:** Results of the estimated values of the parameters of the binary logistic regression model.

Group	Variable	*b*	Standard error of b	Wald chi-square value	*P*	*OR*	Odds ratio with 95% confidence interval
Training group	Gender	0.68	0.33	4.34	0.037	1.97	1.04–3.73
	TI-RADS classification	2.93	0.39	61.83	< 0.001	3.91	1.88–8.14
	Age	-0.07	0.01	36.40	< 0.001	0.93	0.91–0.95
	Maximum diameter of the nodule	0.07	0.03	5.47	0.019	1.07	1.01–1.13
Test group	Gender	-0.07	0.44	2.49	0.115	0.50	0.21–1.18
	TI-RADS classification	3.00	0.57	28.83	< 0.001	3.60	1.35–9.66
	Age	-0.04	0.02	5.93	0.015	0.96	0.94–0.99
	Maximum diameter of the nodule	-0.06	0.04	1.78	0.182	0.95	0.87–1.03

### Image classification based on YOLOv3 model

**Figure 1:**
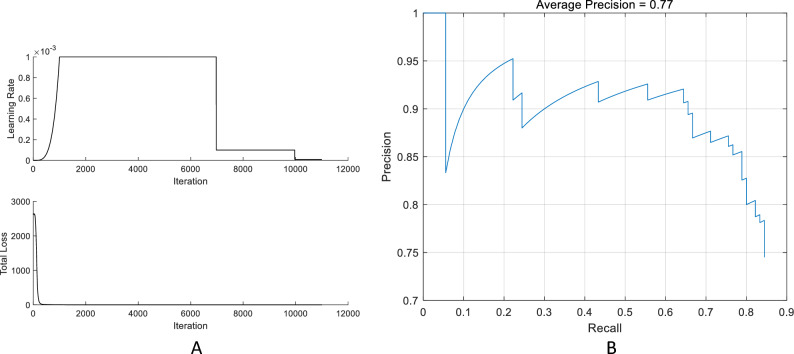
Basic parameters of the YOLOv3 Model. *Note*: (**A**) shows Learning Rate Curve and Loss Curve for the YOLOv3 Model; (**B**) shows Precision-recall curve for the YOLOv3 model.

**Figure 2:**
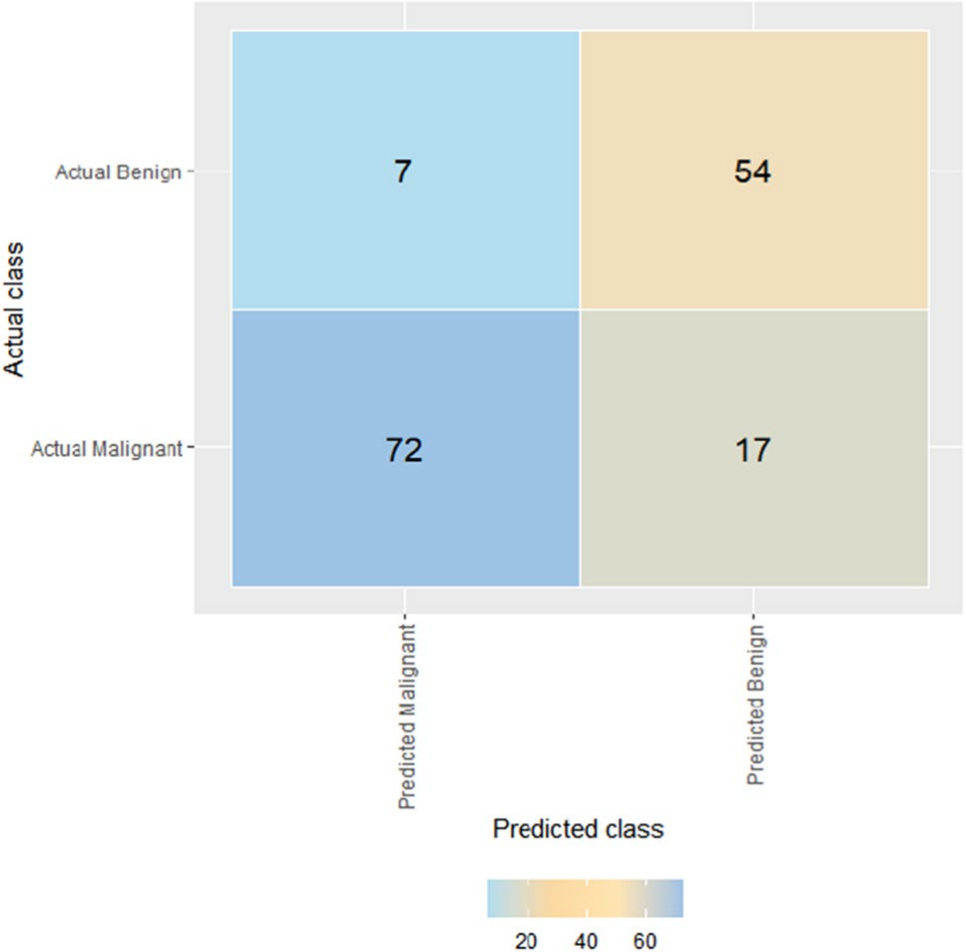
Confusion matrix for the YOLOv3 model.

According to the precision-recall curves under different thresholds, the model achieves relatively accurate classification prediction at different recall levels with an average precision of 0.77 (see Fig. 1). After parameter tuning and training, the effective classification precision of the YOLOv3 model on the test set reaches 0.84. The confusion matrix is presented in Fig. 2. Meanwhile, the AUC value of the model is 0.873 [0.828–0.912] for the training set and 0.836 [0.767–0.905] for the test set.

### Construction of nomogram and performance evaluation

**Figure 3:**
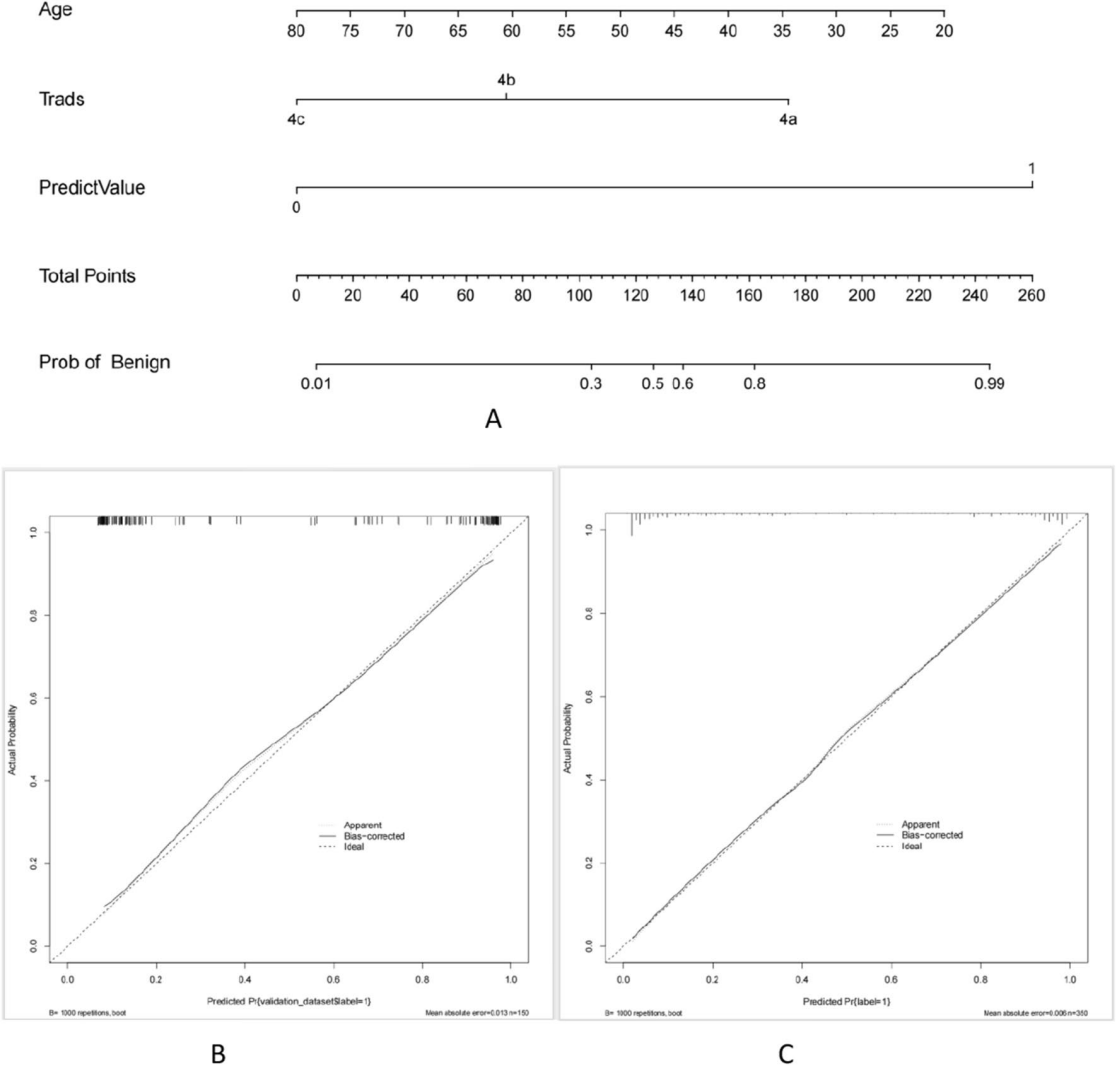
Nomogram for Diagnosing TI-RADS Category 4 Thyroid Nodules. *Note*: (**A**) shows the nomogram for diagnosing the likelihood of benign in TI-RADS category 4 thyroid nodules. The position of each variable on the corresponding axis is solved separately, and a vertical line is drawn on the point axis to represent the number of points; the points of all variables are summed and a line is drawn from the total points axis, and the result is the likelihood of the nomogram diagnosing benignity in TI-RADS category 4 thyroid nodules. (**B**) and (**C**) show the calibration curves of the nomograms in the test group and the training group. The calibration curves depict the calibration of the nomogram. The linear dashed line represents the ideal prediction, the dotted dashed line represents the predictive ability of the nomogram, and the solid line represents the bias correction. The closer the dotted dashed line is to the line dashed line, the higher the prediction accuracy of the nomogram.

**Figure 4:**
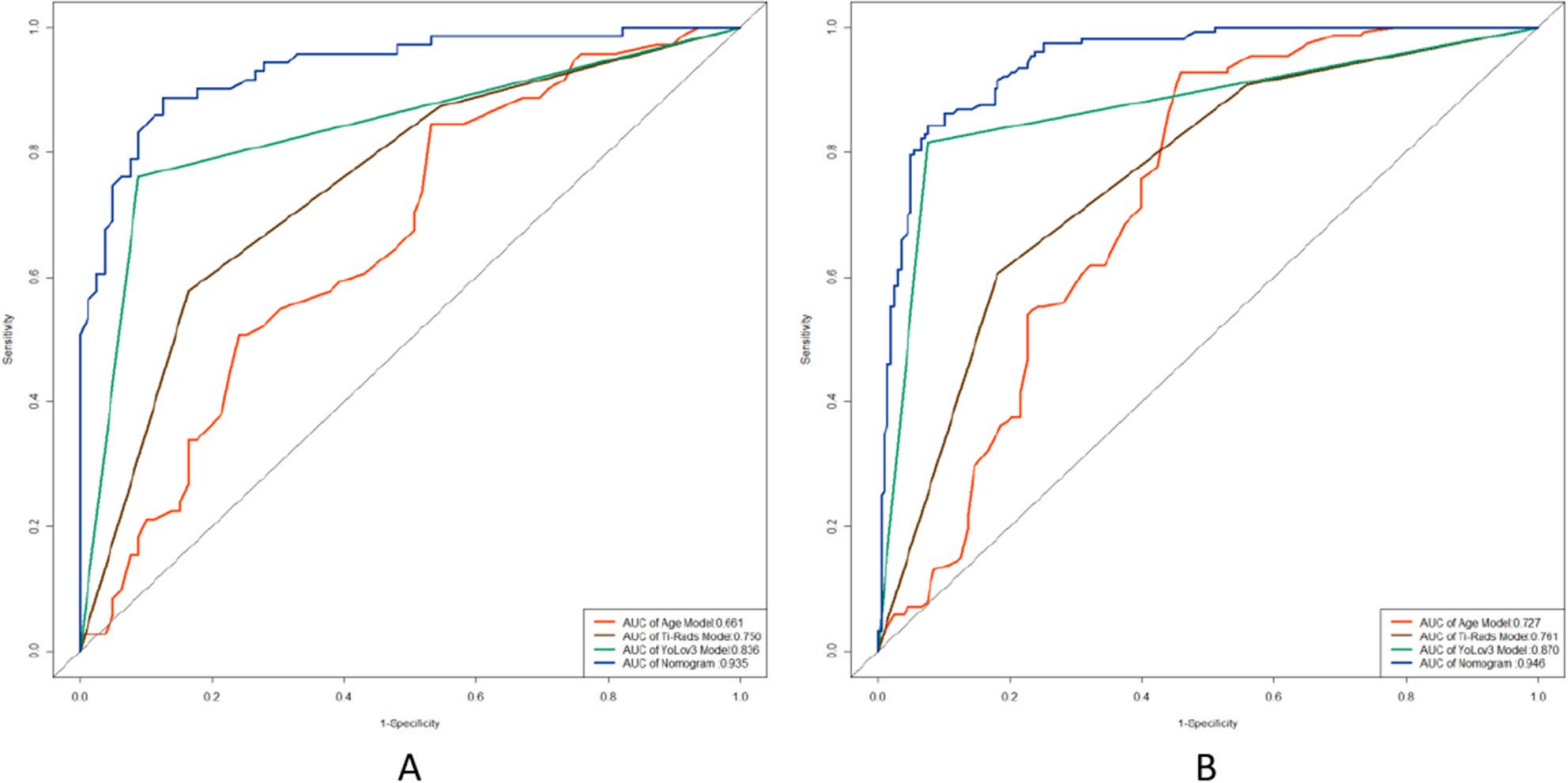
Performance of Nomogram for Diagnosing TI-RADS Category 4 Thyroid Nodules. *Note*: ROC curves for nomogram (blue line), YOLOv3 model (green line), TI-RADS classification (gray line), and age model (red line) in test group (**A**) and training group (**B**).

**Figure 5:**
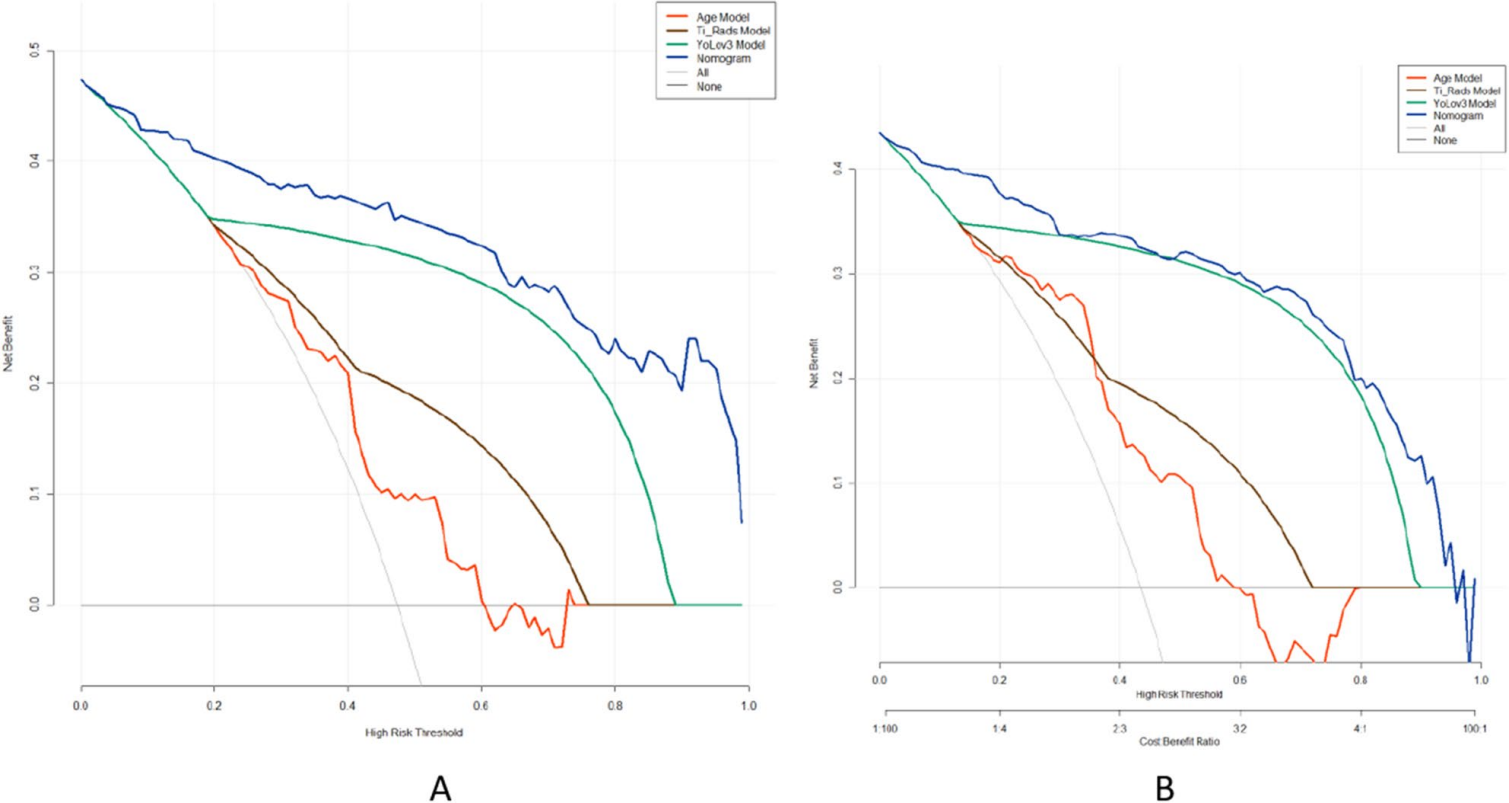
Comparison of the nomogram model and the one-factor model. *Note*: (**A**) and (**B**) are decision curve analyses of the prediction of thyroid cancer by each model in the test and training groups, respectively. y-axis measures net gain. The gray line indicates the assumption that all nodules are malignant (all treatment options). DCA shows that the ability to predict the benign–malignant nature of TI-RADS category 4 thyroid nodules using the nomogram model (blue curve) at any given threshold probability yields more benefit than using a single model. DCA, Decision curve analysis.

The study integrated age, TI-RADS classification, and the YOLOv3 model to construct a nomogram for predicting TI-RADS category 4 thyroid nodules (refer to Fig. 3). The logistic regression algorithm was employed to develop the nomogram, which outperformed other models in terms of AUC values, both in the training and test groups. In the training group, the nomogram achieved an AUC of 0.946, while the YOLOv3 model predicted value had an AUC of 0.870, the TI-RADS classification had an AUC of 0.761, and age had an AUC of 0.727. Similarly, in the test group, the AUC of the nomogram was 0.932, the YOLOv3 model predicted value had an AUC of 0.836, the TI-RADS classification had an AUC of 0.750, and age had an AUC of 0.661. The ROC curves for the training and test groups are depicted in Fig. 4. The Nomogram calibration curves revealed good agreement between the predicted probability of identifying benign and malignant thyroid nodules of TI-RADS category 4 and the actual probability in both the training group (*P* = 0.631, see Fig. 3C) and the test group (*P* = 0.838, see Fig. 3B) (*P* > 0.05). This indicates a high level of reliability and accuracy in the prediction process of the model. Additionally, the performance of the nomogram was evaluated using DCA in both the training and test groups, as detailed in Fig. 5. The DCA assessment consistently demonstrated that the nomogram provided the highest net benefit across the entire threshold range. These findings suggest that the nomogram offers superior results in clinical decision-making, providing a more scientific basis for medical interventions. The results of the nomogram further validate the reliability and utility of the model, offering robust support for optimizing the clinical decision-making process.

## Discussion

Thyroid nodules are a prevalent condition worldwide, with more than 60% of people over 50 years of age having at least one nodule, the majority of which are benign. Ultrasound is a reliable and non-invasive test^9,10^ used to assess the size, morphology, echogenicity, and vascularity. To aid physicians in assessing the risk of thyroid nodules, several classification systems have been proposed and adopted. The most commonly used system is the TI-RADS system, proposed by the American Thyroid Association, which categorizes nodules into TI-RADS 2–5 categories based on their ultrasound performance characteristics^11^. Each category corresponds to a different level of risk and management recommendations. However, TI-RADS category 4 nodules pose a challenge for clinicians as they exhibit suspicious malignant features on ultrasound images but lack obvious highly suspicious malignant features. These nodules may have malignant potential, but a certain percentage of them are ultimately proven to be benign^12^. TI-RADS category 4 nodules can be further subdivided into three subtypes: 4a, 4b, and 4c, with 4c carrying the highest risk of malignancy, where the proportion of cancers may be as high as 80% or more^13^. For TI-RADS 4 nodules, it is generally recommended to perform a puncture biopsy to determine whether the nodule is benign or malignant and to decide whether surgery or other treatments are necessary based on the results. This is because ultrasound results are influenced by several factors, including physician experience, skill, equipment, and the characteristics of the thyroid gland itself. As a result, there may be differences in diagnostic accuracy and efficiency between different physicians, leading to misclassification of some nodules that should be classified as category 3 or 5 as category 4. This misclassification may result in unnecessary puncture biopsy and surgery^14^. Therefore, accurately diagnosing TI-RADS category 4 nodules is a challenge faced by sonographers.

AI is a field of science and technology dedicated to simulating and representing human intelligence using machines^15^. In the field of ultrasound medicine, the development of AI can be traced back to recent decades. Initially, the analysis of ultrasound images relied mainly on traditional image processing methods such as edge detection, filtering, and feature extraction. With the development of machine learning methods based on statistical models and classifiers such as support vector machines and random forests, ultrasound medicine began using these methods for segmentation of ultrasound images, classification, and lesion detection. In recent years, the rapid development of deep learning technology has brought a major breakthrough for ultrasound medicine. Deep convolutional neural networks can learn features directly from original ultrasound images, achieving more accurate lesion detection, segmentation, and diagnosis. Additionally, AI can generate synthetic ultrasound images using techniques such as adversarial networks^16^, which can generate images with different lesion types, scanning parameters, or pathological states. These images are used in education and training, medical research, and image reconstruction. Currently, more and more AI algorithms are being applied in ultrasound equipment to achieve real-time assisted diagnosis. These algorithms provide instant feedback and suggestions during ultrasound scanning, helping doctors perform more accurate lesion localization and assessment. Furthermore, AI can be used in ultrasound medicine for automatic report generation and data analysis. Through natural language processing and data mining methods, key information can be extracted from a large number of ultrasound images and clinical data, generating structured reports to improve work efficiency and the accuracy of clinical decision-making. Although AI has made many advances in the field of ultrasound medicine, it still faces challenges, such as data privacy protection, algorithm interpretability, and clinical acceptability^17^. Therefore, when promoting the application of AI in ultrasound medicine, it is necessary to ensure the safety, reliability, and compliance of the algorithms and work closely with physicians to improve the diagnosis and treatment of ultrasound imaging.

In this study, we employed the YOLOv3 deep learning-based object detection algorithm^18^ to detect TI-RADS category 4 thyroid nodules. This algorithm is renowned for its speed, accuracy, and ease of implementation, allowing for efficient acquisition of a large volume of image information in a short period of time^19^. Additionally, as YOLOv3 is implemented using deep learning methods, the model has the ability to flexibly adapt and optimize to better suit different medical imaging data. The model also supports pre-processing using traditional image processing techniques to further improve diagnostic accuracy and stability. Compared to traditional object detection algorithms, YOLOv3 has faster detection speeds and significant improvements in accuracy. Studies have shown that YOLOv3 is a more advanced and powerful object detection system compared to single-shot detector (SSD) and faster region-based convolutional neural network (Faster R-CNN)^20,21^, with the advantage of real-time modeling over Faster R-CNN^20^. Therefore, using YOLOv3 as the detection model in automatic detection of thyroid nodules has great potential for application.In the medical field, the YOLOv3 model is mainly used for object detection and localization in medical imaging, providing doctors with faster and more accurate methods for disease diagnosis and treatment. Its efficiency and accuracy have been highly evaluated by the medical community and are expected to play an important role in future clinical practice. By using the YOLOv3 model, the efficiency of object detection and localization in medical imaging has been greatly improved, while providing better tools for doctors to improve patient health. The aim of this study is to use the YOLOv3 model to predict the benign and malignant characteristics of TI-RADS category 4 thyroid nodules and validate its effectiveness through experimental results. The experimental results show that the model achieved satisfactory results, with an effective classification accuracy of 84%. To evaluate the performance of the YOLOv3 model in predicting the benign and malignant characteristics of thyroid nodules, we compared and analyzed it with TI-RADS classification and nodule diagnosis results. First, in the training group, we compared the AUC values for predicting benign and malignant TI-RADS category 4 thyroid nodules. The results showed that the AUC of traditional TI-RADS classification diagnosis was 0.761, while the AUC predicted by the YOLOv3 model was 0.870. This indicates that compared to traditional methods, the YOLOv3 model has higher accuracy and performance advantages in predicting the benign and malignant characteristics of thyroid nodules. Additionally, the same comparison was performed in the testing group. The results showed that the AUC of TI-RADS classification diagnosis was 0.750, while the AUC predicted by the YOLOv3 model was 0.836. This means that the YOLOv3 model has relatively high accuracy and robustness in predicting the benign and malignant characteristics of TI-RADS category 4 thyroid nodules, with better predictive performance than traditional methods. Overall, the YOLOv3 model has relatively high accuracy and reliability in predicting the benign and malignant characteristics of TI-RADS category 4 thyroid nodules. This provides doctors with a faster and more accurate tool for disease diagnosis and treatment decision-making, with the potential to improve patient clinical management. However, further research and validation are required to explore and optimize the potential of the YOLOv3 model in medical imaging.

A nomogram is a widely used data visualization tool in medical research and clinical practice^22^. It effectively predicts the probability of a specific event or outcome by transforming the relationships between various variables into a column-line graph. Nomograms are particularly advantageous due to their interpretability and ease of understanding compared to other prediction models. In this study, we constructed a nomogram for predicting the category 4 thyroid nodules in the TI-RADS system by combining the logistic regression algorithm with clinical factors and the YOLOv3 model. The results of our study demonstrated that the nomogram outperformed other models in terms of the AUC in both the training and test groups. This indicates that our nomogram exhibited higher accuracy in predicting the benign and malignant nature of TI-RADS category 4 thyroid nodules, surpassing the predictive ability of models that considered only a single factor.

There are some limitations to this study that need to be addressed. Firstly, the retrospective nature of the study warrants a larger prospective study to validate the reliability of the results before clinical application. Secondly, the study only included data from a single healthcare facility with a relatively small sample size. More ultrasound image data from multiple healthcare facilities are necessary to validate the broad applicability of the results. Finally, the study only analyzed static images, and the quality of ultrasound images is often influenced by the operator's skill level. Static images may not capture dynamically changing malignancy risk characteristics. Therefore, future studies could focus on developing new artificial intelligence models to evaluate dynamic images and improve prediction accuracy.

In conclusion, this study successfully utilized the YOLOv3 model, alongside clinical and ultrasound factors, to accurately classify TI-RADS category 4 thyroid nodules as either benign or malignant. The corresponding nomograms were developed based on the results, significantly improving the accuracy of identifying the benign and malignant nature of these nodules. This comprehensive model combines multiple key factors, allowing for a more comprehensive assessment of nodule characteristics and risk.The visual presentation of the nomogram provides physicians with an intuitive understanding of the predictive results of nodules, facilitating more accurate clinical decision-making. The findings of this study have practical implications for clinical practice. However, to ensure better clinical application, further validation and optimization of the model are necessary, including validation on larger sample sizes and multi-center data, as well as exploring how to incorporate dynamic image information to enhance prediction accuracy.

## Materials and methods

### Study population

This study included a total of 500 patients with TI-RADS category 4 thyroid nodules, who were seen at our hospital between April 2022 and November 2023. The inclusion criteria were as follows: (1) Patients with visible thyroid nodules on ultrasound who underwent biopsy and/or surgical resection. (2) Patients diagnosed with TI-RADS category 4 thyroid nodules based on preoperative ultrasound images by two sonographers with more than 5 years of experience in thyroid ultrasound diagnosis. (3) All nodules underwent puncture biopsy or surgery to obtain pathological results. The exclusion criteria were as follows: (1) Patients with poor-quality ultrasound images. (2) Patients with incomplete clinical and imaging data. (3) Patients who had undergone previous thyroid surgery or other treatments. The study protocol was reviewed by the Medical Ethics Committee of the First Affiliated Hospital of Shandong First Medical University (Qianfoshan Hospital, Shandong Province, China), and the Ethics Committee waived the requirement for informed consent.

### Acquisition and grouping of ultrasound images

Thyroid ultrasonography was performed by our sonographers using ultrasound equipment from different manufacturers, including the GE LOGIQ E9 (probe model ML6-15), the HITACHI ARIETTA 70 (probe model EUP-C715), and the Mindray Resona R9T (probe model L14-5WU). The TI-RADS system^23^, proposed by the American Thyroid Association (ATA), was applied to assess the benign and malignant risk of thyroid nodules. Retrospective diagnosis of all ultrasound images was conducted by two chief physicians from the ultrasound department. In cases where there was a disagreement in the diagnostic results, a more experienced supervisor was consulted for discussion and final agreement. The ultrasound images extracted from the database were saved in JPG format. Clinical and ultrasound data of all study subjects were recorded, including name, age, sex, pathological results or fine-needle aspiration (FNA) results, and ultrasound reports. A total of 500 ultrasound images were included in this study, with 223 cases in the benign group and 277 cases in the malignant group. All images are randomly divided into training and validation groups in a 70%&30%. During the training process, we use the data from the training group for model training and evaluate the performance of the model with the data from the validation group.

### Image processing and data labeling

The JPG format ultrasound images extracted from the database underwent preprocessing steps. First, the images were cropped to retain only the sampling frame, ensuring it matched the size of the original ultrasound image. Additionally, the machine model, machine parameters, and date of examination surrounding the ultrasound image were cropped out to reduce noise and unnecessary information. To annotate the thyroid nodules in the images and assign classification labels, an online annotation tool called https://www.makesense.ai/ was utilized. The tool allowed for precise labeling of the thyroid nodules and setting the labels accordingly. A label of 0 was assigned if the pathological result indicated malignancy, while a label of 1 was assigned for benign results. The annotated images were then saved and exported in yolo format, facilitating further analysis and training of the model.

### Model selection, training, optimization, and validation

In this study, the YOLOv3 (You Only Look Once, Version 3) model was selected due to its suitability for medical images, which often exhibit high resolution, complex anatomical structures, and diverse pathological features. The YOLOv3-based model offers a balance between high detection accuracy and speed, making it well-suited for tasks involving fine lesions or multi-target detection in medical images. The neural network of the chosen model takes input images with dimensions of 256 × 256 pixels. For the loss function, the cross-entropy loss function was selected. The Adam optimization algorithm was employed as the optimizer. A total of 500 training rounds were conducted, with each round iteratively training the model using a batch size of 16. To address the issue of sample imbalance, data augmentation techniques were applied to enhance the training images. These techniques included random rotations, flips, and other operations aimed at expanding the number of samples in certain categories. The YOLOv3 model monitors its training process using two key metrics, learning rate and total loss. The learning rate determines the step size or learning step used by the model to update the parameters in each iteration. On the other hand, total loss quantifies the difference between the model's predicted results and the actual labels, with smaller values indicating a better fit. To evaluate the performance of the binary classification model, we used precision-recall curves at different thresholds. These curves take into account both the classification precision and the recall of the model. Calculating the average precision under the curves provides a comprehensive evaluation. In addition, the performance of the model in the test group can be illustrated by the confusion matrix, which shows the number of true cases (TP), true negatives (TN), false positives (FP) and false negatives (FN). The performance of the model was also evaluated using metrics such as accuracy, recall and AUC.

### Construction of nomograms

Clinical variables were compared between patients with benign and malignant TI-RADS category 4 thyroid nodules using independent samples t-tests for quantitative variables and chi-square or Fisher’s exact tests for qualitative variables. Significant variables identified from the univariate analyses were then used in multivariate logistic regression analyses to predict the degree of malignancy of the TI-RADS category 4 thyroid nodules, followed by the development of a nomogram. The nomogram assigned points based on each included factor, and the total points were used to derive the predicted likelihood of benignity in TI-RADS category 4 nodules. The performance of the nomogram was assessed using ROC curves and AUC values. Calibration of the nomogram was evaluated using calibration curves and the Hosmer–Lemeshow test. Decision Curve Analysis (DCA) was employed to estimate the net benefit at different threshold probabilities.

### Statistical analysis

SPSS (version 26.0), R language (version 4.3), and Matlab (version R2023a) were utilized for statistical analyses. Continuous variables were described as mean ± standard deviation if they followed a normal distribution, and differences between groups were compared using t-tests. If the continuous variables did not follow a normal distribution, they were expressed as median (upper and lower quartiles), and differences between groups were compared using non-parametric rank-sum tests (Mann–Whitney U tests). Categorical variables were expressed as frequencies and percentages, and differences between groups were compared using the chi-square test or Fisher's exact probability method. Logistic regression was used to analyze the risk factors associated with the benign and malignant nature of TI-RADS category 4 thyroid nodules. A *P*-value of less than 0.05 (two-tailed) was considered statistically significant. Nomograms were developed based on the results of the logistic regression model, and their validity was assessed using calibration curves and the Hosmer–Lemeshow test.

### Approval for human experiments

The study was approved by the Clinical Ethics Committee and it was confirmed that all experiments were conducted in accordance with the relevant designated guidelines and regulations.

## Data Availability

The data that support the findings of this study are available from our Ultrasound Clinic but restrictions apply to the availability of these data, which were used under license for the current study, and so are not publicly available. Data are however available from the authors upon reasonable request and with permission of our Ultrasound Clinic.
